# Emergency admission Predictive RIsk Stratification Models: Assessment of Implementation Consequences (PRISMATIC 2): a protocol for a mixed-methods study

**DOI:** 10.3399/BJGPO.2024.0182

**Published:** 2025-02-26

**Authors:** Mark Kingston, Helen Snooks, Alan Watkins, Christopher Burton, Jeremy Dale, Jan Davies, Alex Dearden, Bridie Evans, Bárbara Santos Gomes, Jenna Jones, Rashmi Kumar, Alison Porter, Bernadette Sewell, Emma Wallace

**Affiliations:** 1 Swansea University Medical School, Swansea University, Swansea, UK; 2 School of Health Sciences, University of East Anglia, Norwich, UK; 3 Warwick Medical School, University of Warwick, Warwick, UK; 4 PPI contributor, c/o Swansea University Medical School, Swansea, UK; 5 Swansea Centre for Health Economics (SCHE), Swansea University, Swansea, UK; 6 School of Medicine, University College Cork, Cork, Ireland

**Keywords:** primary health care, health services research, emergency medical services, clinical decision rules

## Abstract

**Background:**

Emergency admissions are costly, increasingly numerous, and associated with adverse patient outcomes. Policy responses have included the widespread introduction of emergency admission risk stratification (EARS) tools in primary care. These tools generate scores that predict patients’ risk of emergency hospital admission and can be used to support targeted approaches to improve care and reduce admissions. However, the impact of EARS is poorly understood and there may be unintended consequences.

**Aim:**

To assess effects, mechanisms, costs, and patient and healthcare professionals’ views related to the introduction of EARS tools in England.

**Design & setting:**

Quasi-experimental mixed-methods design using anonymised routine data and qualitative methods.

**Method:**

We will apply multiple interrupted time-series analysis to data, aggregated at former clinical commissioning group (CCG) level, to look at changes in emergency admission and other healthcare use following EARS introduction across England. We will investigate GP decision making at practice level using linked general practice and secondary care data to compare case-mix, demographics, indicators of condition severity, and frailty associated with emergency admissions before and after EARS introduction. We will undertake interviews (approximately 48) with GPs and healthcare staff to understand how patient care may have changed. We will conduct focus groups (*n* = 2) and interviews (approximately 16) with patients to explore how they perceive that communication of individual risk scores might affect their experiences and health-seeking behaviours.

**Conclusion:**

Findings will provide policymakers, healthcare professionals, and patients, with a better understanding of the effects, costs, and stakeholder perspectives related to the introduction of EARS tools.

## How this fits in

The consequences of using emergency admission risk stratification (EARS) tools in primary care remain unclear. Our previous study found unexpected effects associated with the introduction of EARS tools in South Wales, including increases in emergency admissions to hospital. We will determine if those effects extended across England and investigate how GPs changed practice following introduction of EARS tools. We will also explore patient perspectives, which have been largely overlooked.

## Introduction

Worldwide, discussions on primary healthcare efficiency focus on reorienting systems towards proactive, anticipatory, and integrated care.^1^ This shift responds to rising life expectancy, multimorbidity, and health complexity. Emergency hospital admissions continue to rise despite policy efforts to reduce them.^2^ While potentially lifesaving and preventing long-term morbidity, emergency admissions are generally unwelcome to patients. They are linked to adverse outcomes such as functional decline and hospital-acquired infections. From a provider perspective, these admissions are expensive and constrain planned care.^3^

In the UK, a significant policy response involves the introduction of emergency admission risk stratification (EARS) tools. These tools use routine patient data to generate scores reflecting the risk of emergency hospital admission. Widely implemented in UK general practices^4^ and internationally,^5,6^ EARS tools support targeted approaches to improve care and reduce emergency admissions. The intention, with primary care targeted at those at higher risk, is to prevent many hospitalisations,^2^ notably for ambulatory care sensitive (ACS) conditions such as diabetes, epilepsy, and high blood pressure.^7^ A large variation in ACS admission rates across general practices in England has previously been observed,^8^ driven by factors such as deprivation, multimorbidity, and primary care quality.^9,10^ The introduction of EARS has also been aligned to integrated care approaches and a focus on personalised and holistic care.^11,12^ The UK allocated substantial budgets to EARS initiatives, including more than £480 million for the English Avoiding Unplanned Admissions Enhanced Service (2014–2017).^13^ This service encouraged general practice teams to proactively support patients at high risk of emergency admission following identification using EARS. Further initiatives using EARS have followed across the UK, including the recent roll-out of a new AI tool across South-West England.^14^

Despite such commitments, the impact and worth of EARS as a policy option remains unclear.^12^ It is important to understand the costs and consequences of using risk stratification tools, both beneficial and adverse, to inform future care delivery.^15^

Our previous randomised trial (PRISMATIC) found unexpected effects following EARS introduction to 32 general practices in South Wales, with associated increases in emergency hospital admissions, emergency department (ED) attendances, and days spent in hospital.^16^ Costs to the NHS increased substantially, by an average of £72 per GP-registered patient per year. As a result of PRISMATIC, the planned roll-out of EARS was halted in Wales, saving an estimated £220 million per year, largely through avoided days spent in hospital.^16^

This study builds on the findings of the PRISMATIC trial and responds to the wider EARS literature, including a systematic review.^17,18^ EARS tools have typically been used to identify patients for further intervention (case-finding), often alongside other identification approaches.^19–27^ Overall, there are no high quality studies demonstrating effectiveness, with a limitation being that most comparative studies had used EARS to identify eligible patients in both control and intervention arms, making it challenging to isolate effects. Most studies of EARS have had short follow-up periods (<12 months), although longer-term effects of use are theorised.^28,29^ One US study showed gradual positive effects on admissions, notably for ACS conditions.^30^ Other studies revealed unintended consequences, potentially owing to unmet need,^31^ or lowered hospital admission risk for prioritised patients but not others.^32^

Studies, including qualitative approaches, identify support for tools in principle alongside concerns about model accuracy, data access, and clinical capacity to support patients.^33–35^ Patient perspectives are lacking in the literature.

With ever-increasing demand for emergency and acute care, this research is crucial to determine: (a) if effects found in PRISMATIC extend across England and over a longer period; (b) to understand the mechanisms by which EARS has an effect (intended or unintended); and (c) patient and stakeholder views. PRISMATIC2 employs a ‘natural experiment’ approach to address these aspects.^36^

### Aim

To assess effects, mechanisms, costs, and patient and healthcare professionals’ views related to the introduction of EARS tools in England.

### Objectives

1. Determine the effects of the introduction of EARS tools across all patients and in sub-groups, including those with ACS conditions on emergency admissions, ED attendances, admissions to intensive care units (ICU), time spent (bed days) in hospital and ICU, deaths, and NHS costs.
2. Assess effects of the introduction of EARS tools on clinician behaviour related to admission decisions, including how the threshold for admission and case-mix characteristics change.
3. Describe perspectives of GPs and other practitioners in primary care, ED and working on admission avoidance about use of EARS tools on their management and communication of risk.
4. Capture the views of patients on risk management and how communication of admission risk (scores) may affect their own behaviours, including self-care.

## Method

**Figure 1.**
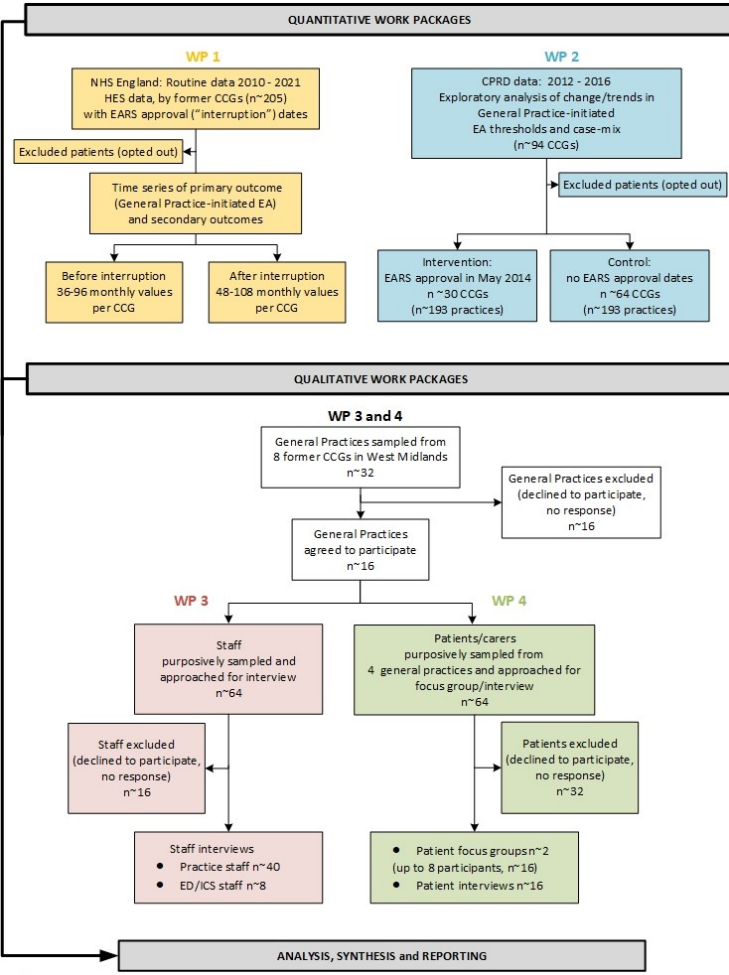
Overview of study design and participant identification.CPRD = Clinical Practice Research Datalink. EA = emergency admission. EARS = emergency admission risk stratification. ED = emergency department. HES = Hospital Episode Statistics. ICS = integrated care system

We will employ a quasi-experimental mixed-methods design to investigate EARS introduction effects, mechanisms, and patient perspectives. Following Medical Research Council (MRC) guidance for development and evaluation of complex interventions,^37^ we will develop a logic model detailing the programme theory with inputs, mechanisms, and intended or unintended effects.^38^ This model will guide four work packages (Figure 1). We will examine processes of EARS adoption using Normalisation Process Theory.^39^

### Quantitative work packages

#### Work package 1 (WP1): Trends in general practice-initiated emergency hospital admissions

We will use multiple interrupted time-series (MITS) analysis^40^ to examine trends in data relating to general practice-initiated emergency hospital admissions in England’s population, aggregated at the former clinical commissioning group (CCG) level, with reference to published dates of EARS approval for use at each CCG (interruption or introduction dates).^41^ With approximately 205 CCGs (study sites) and varying EARS introduction dates, we will analyse routine data from Hospital Episode Statistics (HES), Office for National Statistics (ONS), and Emergency Care Data Set (ECDS) via NHS England. This includes anonymised data on emergency admissions, ED attendances, and hospital and intensive care days at CCG level from 2010–2021, linked to EARS introduction dates.

We will request comprehensive data items, including admission details, demographics, health event specifics, and treatment information. We will aggregate data at study-site level across defined short periods to form time series of our primary outcome (emergency admissions) and secondary outcomes. We will assess data quality across study sites, checking for completeness, unexpected features, or trends. Any anomalies will be explored in the context of site history over the study window, seeking reasons for data 'spikes', omissions, or unexpected variations over time, which may reasonably be attributed to local circumstances or complexities.

We will assess changes associated with EARS introduction, adjusting for demographic and case-mix differences. We will also explore profiles at CCG level, including patient sub-groups of those at highest risk (using frailty risk scores)^42^ and those with ASC conditions; and estimate healthcare resource use costs before and after EARS introduction.

#### Work package 2 (WP2): Changes in thresholds for general practice-initiated emergency hospital admissions

Using individual-level general practice data held within the Clinical Practice Research Datalink (CPRD) repository,^43^ we will explore effects of EARS on thresholds for general practice-initiated emergency hospital admission decisions and the associated case-mix.

We will focus on practices in approximately 30 English CCGs where EARS was approved in May–June 2014, comparing demographic and clinical data for 2 years before and after (at practice and CCG level). We will also link CPRD and HES data, to obtain a more in-depth picture of the effect of the introduction of EARS. We will request equivalent data for a control group of practices within former CCGs (approximately 10) where EARS was approved after June 2016 or not at all.

### Qualitative work packages

We will undertake qualitative work in one English region, recruiting 16 practices across eight CCGs. We will recruit practices using purposive sampling to address diversity in practice size, location, and patient demographics. Experienced qualitative researchers will conduct the interviews and focus groups, which will be recorded and transcribed. We will use Normalisation Process Theory to examine processes of adoption by clinicians.

#### Work package 3 (WP3): Semi-structured interviews with general practice clinical staff

We will interview GPs and other primary care staff involved in emergency admission decision making (approximately 40, ≤3 per practice) to capture views about how introduction and use of the software may have changed perceptions of risk and accountability and emergency admission decision making. We will ask about key inputs, mechanisms, and effects, both intended and unintended. We will also interview ED clinicians and integrated care system (ICS) or former CCG staff with responsibility for admission avoidance (approximately 8) to understand their perspectives on the effect and role of EARS.

#### Work package 4 (WP4): Focus groups and interviews with patients

We will recruit patient participants (approximately 32) from four WP3 practices, selected to account for local demographic diversity. We will explore through focus groups (*n* = 2) and interviews (approximately 16) how patients perceive individual emergency admission risk score communication and its potential impact on experiences and behaviours, including self-care. We will recruit two focus groups, each with up to eight patients, through existing patient networks within the study area. Additionally, we will conduct approximately 16 interviews in person, by phone, or online, with patients recruited via participating practices using letters and telephone follow-up. We will target participants with varied emergency admission experiences, risk profiles, ages, ethnic groups, and long-term conditions. Translation and interpretation will be offered, and participants will receive a £25 incentive gift voucher.

### Data analysis

#### Quantitative

We will calculate site-level measures from patient-level data to summarise and compare sites, with monthly aggregation for primary analysis, and fortnightly aggregation in sensitivity analyses. Outcome measures include rates or number of ED attendances, general practice-initiated emergency admissions, and proportions of re-attendances and inpatient admissions. We will profile patient demographics and use International Classification of Diseases, Tenth Revision (ICD10) codes for diagnoses to explore modal causes of attendance and identify sub-groups (for example, patients identified with ACS conditions). Exploratory time-series methods will evaluate trends pre- and post-EARS introduction, considering seasonal patterns and outliers. Two time periods starting in 2010 will be analysed: one including the COVID-19 pandemic period to 2021; and a pre-pandemic one ending on 1 March 2020.

Analysis of MITS models will test the null hypothesis that the introduction of EARS has no effect on the trend in outcome measures. We will have at least 120 monthly values for any outcome. Based on assessment of HES accident and emergency data within this period, we expect moderate autocorrelation, in the range of 0.2–0.5, for a lag of 1 month. Interpolating from available data, we should, using 90% power and 5% significance, be able to detect an effect of size in the range of 0.5–0.8 for a single series; with concomitantly greater power in analysing panel data.

Planned sensitivity analyses will assess: the robustness of findings for different aggregation periods and frailty risk thresholds; and consistency of findings across pre-specified patient sub-groups.

We will undertake a cost consequences analysis (CCA) alongside the clinical effectiveness analysis. We will estimate healthcare resource use and use weighted standard unit costs applied to resource use data based on Healthcare Resource Group (HRG) codes, to calculate the cost associated with healthcare use before and after introduction of EARS. We will explore costs for the total study population and sub-groups and present disaggregated resources, their unit costs, and a range of outcomes together with estimates of mean costs with appropriate measures of variation. Our primary CCA will be supplemented by sensitivity analyses, to account for uncertainty in parameter estimates. Discounting will be applied at the standard rate where follow-up periods exceed 1 year. Implementation costs per patient will be based on extrapolation from PRISMATIC.^17^

For WP2, we will analyse linked demographic, case-mix and clinical data from CPRD and HES to explore thresholds for emergency admission decisions before and after EARS introduction and compare with the control group data.

#### Qualitative

We will follow a framework approach for qualitative data analysis,^44,45^ informed by the logic model and Normalisation Process Theory. We will convene a qualitative sub-group of researchers, clinicians, and patient and public contributors. The sub-group will review transcripts and develop codes, and an initial analytical framework for testing and revision with both patient and health professional datasets. Each transcript will be reviewed by a minimum of two members. The sub-group will discuss interpretation and emerging themes and consider any contradictions or inconsistencies. Analysis will take place first within and then across groups (health professionals; patients). Findings will be structured around themes with verbatim quotations. Findings will be grounded in first-hand accounts of technology introduction and impact and have transferability to other settings and healthcare technologies. The sub-group will use the findings to refine the logic model.

### Public and patient involvement (PPI)

We are strongly committed to the involvement of patients and the public, and the UK Standards for Public Involvement will be followed.^46^ Two patient/public contributors are co-applicants and sit on the research management group and a further two on an independent steering committee. Patient/public contributors were involved in research development.

In developing this study we held a focus group with a patient involvement group,^47^ and found support for using EARS tools to frame discussion of risk based on shared decision-making principles.^48^

### Synthesis and dissemination

We will formally synthesise qualitative and quantitative data, sequentially, using a triangulation protocol described by O’Cathain *et al* and the analytical approach outlined by Östlund *et al*.^49,50^ We will develop communication, publication, and dissemination plans to inform our wider engagement activities. We will share findings widely in partnership with policymakers, health service providers, and patient and public contributors and participants.

## Discussion

### Summary

PRISMATIC2 builds on our previous evaluation of a tool in one Health Board area (PRISMATIC), by evaluating the implementation of EARS software across England.

### Strengths and limitations

Our main study strength is in the use of mixed methods that address national- and site-level data and perspectives. The MITS approach is particularly suited to evaluating interventions introduced at a population level over a defined period that target population level health outcomes, while site-based qualitative data will provide important insights from practitioners and patients. A potential limitation lies with undertaking qualitative work in one geographic region. However, we will recruit across multiple former CCGs and practices with varying sociodemographic patient profiles, and our analytic approach is designed to produce generalisable findings.

### Implications for research and practice

This study will provide policymakers, practitioners, and the research community with a better understanding of the effects of predictive risk software on costs, processes, and outcomes of care across a range of settings.

## Research Registration

Research Registry Unique Identifying Number: researchregistry8491

https://www.researchregistry.com/browse-the-registry#home/registrationdetails/63738f3f6c6999002195f8c9/

## Data

NHS England and CPRD data is available with appropriate research governance approvals. Qualitative data is not available due to study specific consent arrangements.

## References

[1] Department of Health and Social Care (2021). Integration and innovation: working together to improve health and social care for all.

[2] Santos R, Rice N, Gravelle H (2020). Patterns of emergency admissions for ambulatory care sensitive conditions: a spatial cross-sectional analysis of observational data. BMJ Open.

[3] Steventon A, Deeny S, Friebel R (2018). Emergency hospital admissions in England: which may be avoidable and how?.

[4] Kingston M, Griffiths R, Hutchings H (2020). Emergency admission risk stratification tools in UK primary care: a cross-sectional survey of availability and use. Br J Gen Pract.

[5] Mora J, Iturralde MD, Prieto L (2017). Key aspects related to implementation of risk stratification in health care systems—the ASSEHS study. BMC Health Serv Res.

[6] Khanna S, Rolls DA, Boyle J (2019). A risk stratification tool for hospitalisation in Australia using primary care data. Sci Rep.

[7] Purdy S, Griffin T, Salisbury C, Sharp D (2009). Ambulatory care sensitive conditions: terminology and disease coding need to be more specific to aid policy makers and clinicians. Public Health (Fairfax).

[8] Busby J, Purdy S, Hollingworth W (2017). Opportunities for primary care to reduce hospital admissions: a cross-sectional study of geographical variation. Br J Gen Pract.

[9] Busby J, Purdy S, Hollingworth W (2015). A systematic review of the magnitude and cause of geographic variation in unplanned hospital admission rates and length of stay for ambulatory care sensitive conditions. BMC Health Serv Res.

[10] O’Cathain A, Knowles E, Maheswaran R (2014). A system-wide approach to explaining variation in potentially avoidable emergency admissions: national ecological study. BMJ Qual Saf.

[11] NHS England (2015). Using case finding and risk stratification: a key service component for personalised care and support planning.

[12] NHS England Operational Research and Evaluation Unit (2017). New care models: risk stratification: learning and impact study.

[13] NHS England (2014). Enhanced service specification: avoiding unplanned admissions: proactive case finding and patient review for vulnerable people. http://www.england.nhs.uk/wp-content/uploads/2014/08/avoid-unplanned-admissions.pdf.

[14] NHS England (2023). AI to help South West NHS spot people at risk of emergency admission. https://www.england.nhs.uk/south/2023/12/05/ai-to-help-south-west-nhs-spot-people-at-risk-of-emergency-admission/.

[15] Lewis G (2015). Next steps for risk stratification in the NHS.

[16] Snooks H, Bailey-Jones K, Burge-Jones D (2019). Effects and costs of implementing predictive risk stratification in primary care: a randomised stepped wedge trial. BMJ Qual Saf.

[17] Snooks H, Bailey-Jones K, Burge-Jones D (2018). Predictive risk stratification model: a randomised stepped-wedge trial in primary care (PRISMATIC).

[18] Kingston MR, Evans BA, Nelson K (2016). Costs, effects and implementation of routine data emergency admission risk prediction models in primary care for patients with, or at risk of, chronic conditions: a systematic review protocol. BMJ Open.

[19] Abell J, Hughes J, Reilly S (2010). Case management for long-term conditions: developing targeting processes. Care Manag J.

[20] Baker A, Leak P, Ritchie LD (2012). Anticipatory care planning and integration: a primary care pilot study aimed at reducing unplanned hospitalisation. Br J Gen Pract.

[21] Dhalla IA, O’Brien T, Morra D (2014). Effect of a postdischarge virtual ward on readmission or death for high-risk patients: a randomized clinical trial. JAMA.

[22] Freund T, Mahler C, Erler A (2011). Identification of patients likely to benefit from care management programs. Am J Manag Care.

[23] Hall S, Kulendran M, Sadek A-R (2011). Variability in selecting patients to manage in the community: a service evaluation of community matron’s case-finding strategies. Fam Pract.

[24] Levine S, Steinman BA, Attaway K (2012). Home care program for patients at high risk of hospitalization. Am J Manag Care.

[25] McEvoy P, Escott D, Bee P (2011). Case management for high-intensity service users: towards a relational approach to care co-ordination. Health Soc Care Community.

[26] Reilly S, Abell J, Brand C (2011). Case management for people with long-term conditions: impact upon emergency admissions and associated length of stay. Prim Health Care Res Dev.

[27] Department of Health (2009). Supporting people with long term conditions: commissioning personalised care planning — a guide for commissioners.

[28] Stokes J, Shah V, Goldzahl L (2021). Does prevention-focused integration lead to the triple aim? An evaluation of two new care models in England. J Health Serv Res Policy.

[29] Dorr DA, Ross RL, Cohen D (2021). Primary care practices’ ability to predict future risk of expenditures and hospitalization using risk stratification and segmentation. BMC Med Inform Decis Mak.

[30] Kodner DL (2015). Managing high-risk patients: the Mass General care management programme. Int J Integr Care.

[31] Stokes J, Kristensen SR, Checkland K (2017). Does the impact of case management vary in different subgroups of multimorbidity? Secondary analysis of a quasi-experiment. BMC Health Serv Res.

[32] Soto-Gordoa M, de Manuel E, Fullaondo A (2019). Impact of stratification on the effectiveness of a comprehensive patient-centered strategy for multimorbid patients. Health Serv Res.

[33] Evans BA, Dale J, Davies J (2022). Implementing emergency admission risk prediction in general practice: a qualitative study. Br J Gen Pract.

[34] Freund T, Wensing M, Geissler S (2012). Primary care physicians’ experiences with case finding for practice-based care management. Am J Manag Care.

[35] Kingston MR (2024). A multi methods study of emergency admission risk stratification in primary care [PhD Thesis, Swansea University, Cronfa repository].

[36] Craig P, Cooper C, Gunnell D (2012). Using natural experiments to evaluate population health interventions: new Medical Research Council guidance. J Epidemiol Community Health.

[37] Skivington K, Matthews L, Simpson SA (2021). A new framework for developing and evaluating complex interventions: update of Medical Research Council guidance. BMJ.

[38] Mills T, Lawton R, Sheard L (2019). Advancing complexity science in healthcare research: the logic of logic models. BMC Med Res Methodol.

[39] May C, Finch T (2009). Implementing, embedding, and integrating practices: an outline of Normalization Process Theory. Sociology.

[40] Hudson J, Fielding S, Ramsay CR (2019). Methodology and reporting characteristics of studies using interrupted time series design in healthcare. BMC Med Res Methodol.

[41] NHS England (2023). List of risk stratified approved organisations 2023..

[42] Hollinghurst J, Housley G, Watkins A (2021). A comparison of two national frailty scoring systems. Age Ageing.

[43] Herrett E, Gallagher AM, Bhaskaran K (2015). Data resource profile: Clinical Practice Research Datalink (CPRD). Int J Epidemiol.

[44] Ritchie J, Lewis J, McNaughton Nicholls C, Ormston R (2013). Qualitative research practice: a guide for social science students and researchers.

[45] Gale NK, Heath G, Cameron E (2013). Using the framework method for the analysis of qualitative data in multi-disciplinary health research. BMC Med Res Methodol.

[46] UK Standards for Public Involvement (2019). Better public involvement for better health and social care research.

[47] Evans BA, Gallanders J, Griffiths L (2020). Public involvement and engagement in primary and emergency care research: the story from PRIME Centre Wales. Int J Popul Data Sci.

[48] Kingston MR, Davies J, Snooks H (2024). Patient perspectives on the acceptability of emergency admission risk prediction: A focus group study. Ann Public Health Reports.

[49] O’Cathain A, Murphy E, Nicholl J (2010). Three techniques for integrating data in mixed methods studies. BMJ.

[50] Östlund U, Kidd L, Wengström Y, Rowa-Dewar N (2011). Combining qualitative and quantitative research within mixed method research designs: a methodological review. Int J Nurs Stud.

